# Supplementary material for: Efficacy of Cytoreductive Surgery (CRS) + HIPEC in Gastric Cancer with Peritoneal Metastasis: Systematic Review and Meta-Analysis
Source: Cancers (Basel). 2024 May 18;16(10):1929. doi: 10.3390/cancers16101929 (PMC11119026; doi:10.3390/cancers16101929)
Supplement: Supplementary file 1 [file cancers-16-01929-s001.zip › cancers-2970274-supplementary.pdf]

Author, Year	N CRS + HIPEC	Neoadjuvant chemotherapy	TRIPLETE	FOLFOX	FLOT	Others
**Yang, 2011**	34	NA	NA	NA	NA	NA
**Yarema, 2014**	20	0	NA	NA	NA	NA
**Wu, 2015**	11	0	NA	NA	NA	NA
**H-T Wu, 2016**	38	28 (56)	NA	NA	NA	NA
**Boerner, 2016**	50	NA	NA	NA	NA	NA
**Chia, 2016**	81	39 (48)	NA	NA	NA	NA
**Topal 2017**	32	30	30	0	0	0
**Caro, 2018**	35	35 (100)	NA	NA	NA	NA
**Kim, 2018**	38	31	NA	NA	NA	NA
**Manzanedo, 2019**	88	84	NA	NA	6	78
**Bonnot, 2019**	180	113 (62.8)	NA	NA	NA	NA
**Rau, 2019**	58	57 (98)	13	NA	35	9
**Rau, 2020**	235	174 (74)	NA	NA	NA	NA
**Zhong-He Ji, 2020**	125	44 (35)	NA	NA	NA	NA
**Rosa, 2021**	23	38 (44.7)	NA	NA	NA	NA
**Bagdwell, 2021**	20	20 (100)	3	17	0	0
**Marano 2021**	91	60 (65.9)	NA	NA	NA	NA
**Somashekhar, 2022**	16	6	NA	NA	NA	NA
**Santullo, 2023**	20	20 (100)	0	13	7	0
**Buckarma, 2023**	22	22 (100)	NA	NA	NA	NA
**Green, 2023**	41	41 (100)	NA	23	10	8
**Rau, 2023**	59	28	NA	NA	NA	NA
**Allievi, 2023**	27	23 (85.2)	11	6	1	5
**Kobialka, 2023**	25	24 (99)	14	0	10	0

Supplementhary Matherial: Neoadjuvant therapies and regimen were summarized.
FOLFOX: 5-Fluorouracile + Oxaliplatino; FLOT: 5-Fluorouracile + Oxaliplatino + Docetaxel; TRIPLETE: ECF (Epirubicin + Cisplatin + 5-Fluorouracile) or ECX (epirubicin, cisplatin, and capecitabine).
